# 3D Printing of Fe_27_Al_24_Ni_22_Cu_18_Co_9_ High-Entropy Alloy Scaffold with Direct Ink Writing for the Degradation of Methyl Red Azo Dye

**DOI:** 10.1021/acsomega.5c06103

**Published:** 2025-10-30

**Authors:** Oriol Rius-Ayra, Alisiya Biserova-Tahchieva, Marina Carmona-Ruiz, Núria Llorca-Isern

**Affiliations:** CMQF Departament de Ciència dels Materials i Química Física, 16724 Universitat de Barcelona, Martí i Franquès 1-10, 08028 Barcelona, Spain

## Abstract

Dyes are emerging pollutants that can be classified in various ways, with azo dyes being among the most persistent in water. These dyes contain conjugated double bonds and azo groups (R–N=N–R’), making their degradation in water a significant challenge. In this study, we demonstrate that a high-entropy alloy (HEA) with the composition Fe_27_Al_24_Ni_22_Cu_18_Co_9_ can be synthesized using mechanical alloying and subsequently 3D printed using material-extrusion direct ink writing. X-ray diffraction, transmission electron microscopy, and field emission scanning electron microscopy confirmed the synthesis of a new HEA with a face-centered cubic crystal structure after 40 h of mechanical alloying. The degradation properties of the 3D-printed HEA were then investigated for the azo dye known as methyl red, revealing its efficiency at 60 °C in the presence of nitric acid (pH = 1.50), with a degradation time of 28 min and an efficiency of 99%. Moreover, according to the results of high-performance liquid chromatography, we propose the degradation mechanism occurring at the surface of the Fe_27_Al_24_Ni_22_Cu_18_Co_9_ HEA that effectively degrades methyl red and leading to the cleavage of the azo bond and generating two different products.

## Graphical abstract

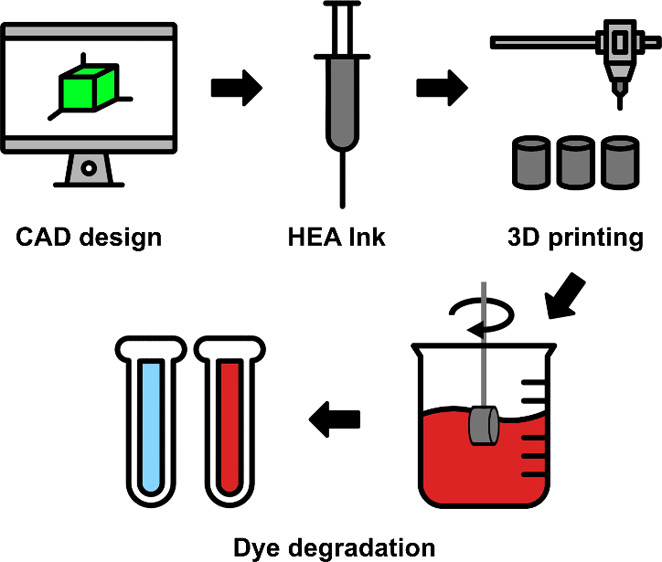

## 1 Introduction

Nowadays, a new category of contaminants known as emerging pollutants has been identified. These are defined as compounds that are not currently regulated by existing water-quality standards, have not been extensively studied, and are considered potential threats to ecosystems, human health, and safety.^1^ This group typically includes pharmaceutical compounds, dyes, oils, and microplastics, among others. Due to their widespread use and chemical stability, they tend to persist in the environment.^1,2^ In case of dyes, they can be classified in different manners^3^ but one of the dyes family that is the most persistent in water are the azo dyes. These compounds present conjugated double bonds as well as azo groups (R–N=N–R’) which make them persistent in water. These dye groups represent over 50% of all commercial dyes, but on the contrary, they are not the most common pollutants to be studied as they just represent 9.33% of the published articles.^4^ The reason why they are not completely studied is because of their great stability and the difficulty to degrade them. In fact, some attempts have been made such as the use of nickel­(II) oxide nanoparticles for the removal process of methyl orange dye,^5^ Co-based metallic glass (Co_78_Si_8_B_14_) to remove acid orange II,^6^ or generating oxygen reactive species with titanium dioxide (TiO_2_) to degrade methyl orange,^7^ among others.^8–10^ In this scenario, additive manufacturing (AM) can improve the scalability of materials because of their rapid prototyping and use 3D-printed materials to degrade this kind of dye from water.

3D printing also known as AM has led to printing different materials for a wide variety of applications such as the use of polylactic acid for bone repair,^11^ a metal–organic framework (MOF) for the simultaneous removal of hydrocarbons and dyes,^12^ or 3D scaffolds based on n-TiO_2_ for photocatalytic methyl orange degradation,^13^ among others.^14–16^ Recently, metals and alloys in additive in manufacturing have attracted the attention because of a wide verity of applications such as the ammonia electrosynthesis with 3D-printed copper electrodes,^17^ the design of chemical reactors and catalysts made of Co, Ni, and Fe for direct conversion of molecules (CO, CO_2,_ and CH_4_) into liquid fuel and syngas,^18^ or the design of impregnated palladium on silica monolith for the Sonogashira and Suzuki reactions.^19^

Despite these applications, advanced metallic materials like HEAs, which show interesting environmental applications,^20–22^ are difficult to in situ obtain 3D-printed structures due to their characteristics.^23–25^ In fact, just a few AM technologies like powder bed fusion (PBF) and direct energy deposition (DED) systems allow one to directly print HEAs, but both technologies are characterized by their fast heating and fast cooling rates.^26–28^ This phenomenon does not allow the obtaining of metastable materials such as some kinds of HEAs because the heating and cooling rates would cause diffusion of metals and consequently the loss of a solid solution. Fortunately, HEAs can be obtained with other processing technologies like mechanical alloying (MA)^29,30^ that allows to achieve nonequilibrium alloys from elemental metallic powders showing a high variability of crystal structures, enhanced properties as well as provide a wide screening of HEA composition.^31–34^ As metastable structures of HEAs cannot be heated because the microstructure could change and then cause a loss of the advanced functional properties, an AM technology that does not involve high temperatures or solid melting is used. For this reason, it is important to consider material-extrusion direct ink writing (ME-DIW), a versatile and effective 3D printing technology that enables the fabrication of three-dimensional geometries through the extrusion and layer-by-layer deposition of high-viscosity ink containing solid particles mixed with a polymeric binder.^35^ This technique allows for 3D printing without heating of the extruded ink. Consequently, ME-DIW can be used to print HEAs, preserving their nonequilibrium structure and maintaining their enhanced properties.

Herein, we demonstrate that a nonequilibrium high-entropy alloy (HEA) with the composition Fe_27_Al_24_Ni_22_Cu_18_Co_9_ can be synthesized using MA and subsequently 3D printed using ME-DIW, enabling the fabrication of scaffolds through extrusion and layer-by-layer deposition of a high-viscosity ink containing metallic particles. Characterization techniques including X-ray diffraction (XRD), transmission electron microscopy (TEM), and field emission scanning electron microscopy (FESEM) confirm the achievement of a new Fe_27_Al_24_Ni_22_Cu_18_Co_9_ HEA with a face-centered cubic (FCC) crystal structure after 40 h of MA. Subsequently, the degradation properties of the 3D-printed HEA for degrading methyl red were investigated, revealing its efficiency at 60 °C in the presence of nitric acid (pH = 1.50), with a degradation time of 28 min.

## 2 Materials and Methods

HEAs can be defined by their configurational entropy (Δ*S*_conf_) at high temperature when Δ*S*_conf_ ≥ 1.5*R* (*R* = 8.314 J mol^–1^ K^–1^) which is the entropy inherent to the alloy. Moreover, the thermodynamic parameter (Ω ≥ 1.1) and difference in atomic radii (δ ≤ 6.6) are required for the obtention of a solid solution.^36^ Then, the atomic ratio of the alloys was precisely studied, and the Ω and δ parameters were calculated to predict the solid solution formation and determine the optimal proportions of each element (Table 1).

**Table 1:** Composition (% at), Thermodynamic Parameter (Ω), and Difference in Atomic Radii (δ) for the Prepared HEA

sample	composition (% at)	Ω	δ
HEA	Fe_27_Al_24_Ni_22_Cu_18_Co_9_	3.0	6.8

### 2.1 Preparation of the HEA

The HEA was synthesized from commercial powders of elemental Fe (99.5%), Cu (99.5%), Co (99.5%), Al (99.5%), and Ni (99.5%), all purchased from Scharlau. The elemental metallic powders underwent MA for durations ranging from 1 to 40 h at 300 rpm, using a Fritsch Pulverisette 6 high-energy planetary ball miller, operated at room temperature. The ball-to-powder weight ratio was maintained at 20:1, employing high-strength steel balls with a diameter of 12 mm and high-strength steel vials. Prior to milling, argon (>99.9996%, purchased from Linde) was introduced to create an inert atmosphere and prevent oxidation of the metallic particles during the MA process. Samples were collected within a glovebox under an argon atmosphere to maintain inert conditions. To prevent carbon contamination, no process control agent was added to the initial powder mixture.

### 2.2 3D Printing

The desired structures have been printed using a direct ink writing (PowerDIW) 3D printer designed and manufactured by the CIM UPC Foundation (CIM UPC). The 3D printer is equipped with a high-force extrusion head (>820 N), capable of printing highly viscous inks or pastes containing powdered HEA and organic binders. To prepare the ink, 1.5 g of polylactic acid (PLA Ingeo 4032 purchased from NatureWorks LLC) was dissolved in 4 g of tetrahydrofuran (THF purchased from Sigma-Aldrich) and left to dissolve for 10 min to ensure homogenization. The HEA paste was prepared by mixing 9 g of HEA powder (97 ± 5 μm) with the polymer solution (weight ratio of 3.4:1), using a ball mill mixer (8000 M Mixer/Mill, SPEX SamplePrep) for 15 min. Before printing the final structure, different geometries such as solid cubes (5 mm × 5 mm × 5 mm) and different printing parameters were tested (Supporting Information: Additional printing details) until a scaffold structure was achieved with a height of 8 mm, a diameter of 14 mm, a wall thickness of 2 mm, an infill of 20, and 85% of metallic particles (Figure 1). Table 2 shows the optimal printing parameters obtained for the developed HEA pastes.

**Figure 1:**
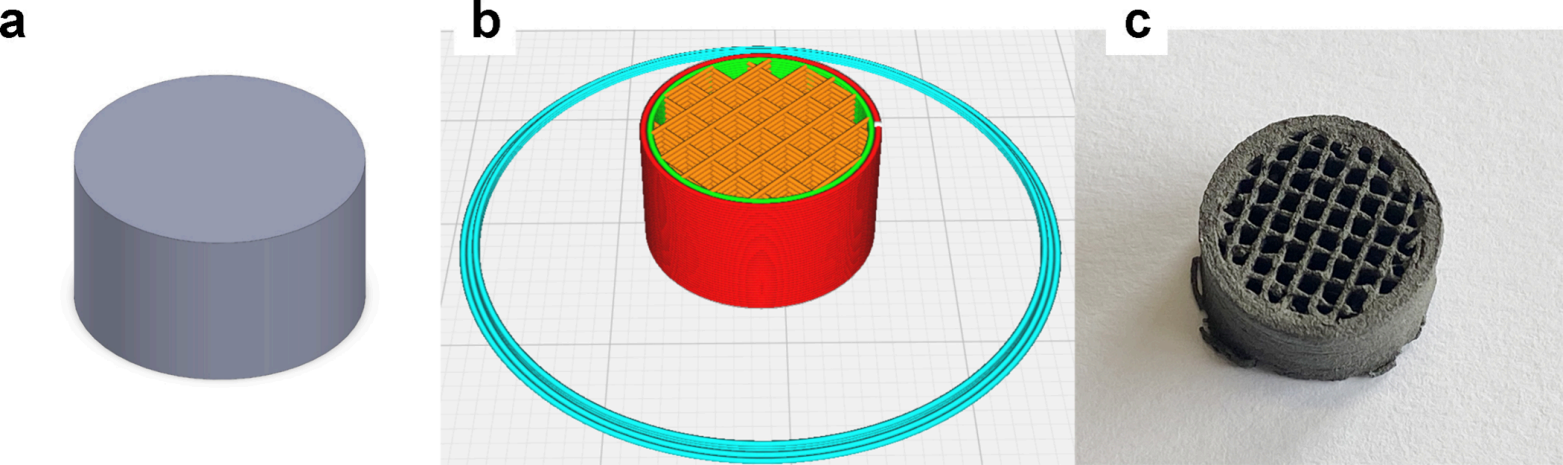
Process design of the achieved 3D-printed HEA: (a) parametric design, (b) 3D printing conditions, and (c) 3D-printed HEA.

**Table 2:** Optimal Printing Parameters Obtained for the Developed HEA Inks

nozzle size (mm)	layer height (mm)	extrusion width (mm)	printing temperature (°C)	bed temperature (°C)	printing speed (mm/s)	volumetric flow rate (mm^3^/s)
0.8	0.3	0.88	25	25	12	3.5

### 2.3 Characterization Techniques

A JEOL J-7100 Field Emission scanning electron microscope with an energy dispersive spectroscopy (EDS) detector was used to characterize the particle morphology and the semiquantitative analysis as well. XRD was carried out using a PANalyticalX’Pert PRO MPD q/q Bragg–Brentano powder diffractometer of 240 mm radius with the Co Kα radiation (1.7903 Å). The phases of the achieved powders after MA were identified by X’PertHighScore Plus software. High-resolution transmission electron microscopy (HRTEM) and selected area electron diffraction (SAED) were conducted by using a JEOL JEM 2100 microscope coupled to an EDS detector. Single Crystal 5 software was used to determine the interplanar distances and planes. Attenuated total reflectance Fourier transform infrared spectroscopy (ATR-FTIR) was used in the range of 4000–525 cm^–1^ at a resolution of 4 cm^–1^ and accumulation of 32 scans with Thermo Scientific Nicolet iZ10, ATR diamond, and detector DTGS. Brunauer–Emmett–Teller (BET) was applied to calculate the specific surface area and the pore size on the basis of nitrogen adsorption isotherm measurements at 77 K with a TriStar 3000 V 6.04 A in a relative pressure (*P*/*P*_o_) range from 0.011 to 0.349. The degradation of the azo dye was carried out by Specord 205, Analytik Jena, UV–vis spectrophotometer in the range between 200 and 800 nm, at a speed of 10.0 nm/s, integration time of 0.10 s, and 1.0 nm of delta lambda with extractions carried on every minute until the total decolorization at different temperatures. Precision cells made of quartz SUPRASIL were used with a light path of 10 mm. A blank of Mili-Q water was used in order to calibrate the UV–vis spectrophotometer. The analysis of the dye degradation products was performed using high-resolution high-performance liquid chromatography mass spectrometry (HR-HPLC-MS) with an LTQ-Orbitrap Velos mass spectrometer (Thermo Fisher Scientific), equipped with an ion trap and Fourier transform analyzer, coupled to an Ultimate 3000 UHPLC chromatograph (Thermo Fisher Scientific). Prior to analyzing the degradation products, a Milli-Q water blank was run, and samples were measured in triplicate.

### 2.4 Degradation of Azo Dye

Methyl red (C_15_H_15_N_3_O_2_), an azo compound, was employed to assess the degradation capability of the HEA in 200 mL of aqueous solution (10 mg/L of methyl red). Different temperatures (20, 40, and 60 °C) and pH levels (pH = 1.5, pH = 7, and pH = 14) were investigated as potential factors affecting degradation in preliminary results. First, it was observed that methyl red remained stable at 60 °C over extended periods with and without the presence of nitric acid, indicating that temperature alone does not cause degradation. No significant changes were observed at 20 or 40 °C; however, a color change was noted at 60 °C, which may be related to partial degradation of methyl red. In contrast, neither neutral nor alkaline pH conditions led to degradation, highlighting the importance of an acidic environment to provide protons to the aqueous solution, as will be further discussed. Subsequently, the 3D-printed HEA was introduced into the dyed solution with a pH of 1.5, prepared using 65% (w/w) extra-pure HNO_3_ (purchased from Scharlau), under stirring. Throughout the decolorization process, 5 mL aliquots were withdrawn at different time intervals to monitor color changes via UV–vis spectrophotometry. The degradation process was repeated three times.

## 3 Results and Discussion

### 3.1 Characterization

The FESEM micrographs showed a consistent and homogeneous morphology of the prepared HEA with a particle size distribution of 97 ± 5 μm (Figure 2). Notably, during the high-energy ball milling (HEBM) process, metallic particles tend to agglomerate and disperse, particularly with increased rotational speed. Following the printing of the HEA using ME-DIW, the resulting structure exhibited layers and voids corresponding to the computer-aided design (CAD) (Figure 2b). Additionally, the micrographs revealed the presence of both HEA metallic particles and the polymeric binder used in preparing the composite ink. Upon closer examination at higher magnification, the rounded morphology of the HEA particles embedded in the polymeric binder became evident (Figure 2c). Furthermore, the compositional map generated by EDS demonstrated the homogeneous distribution of the five elements comprising the HEA (Fe_27_Al_24_Ni_22_Cu_18_Co_9_) throughout the metallic particles, corresponding to the Kα lines for each element. Carbon, conversely, was detected in the polymeric matrix of the binder as well as in the cold-mounting resin (Figure 2d).

**Figure 2:**
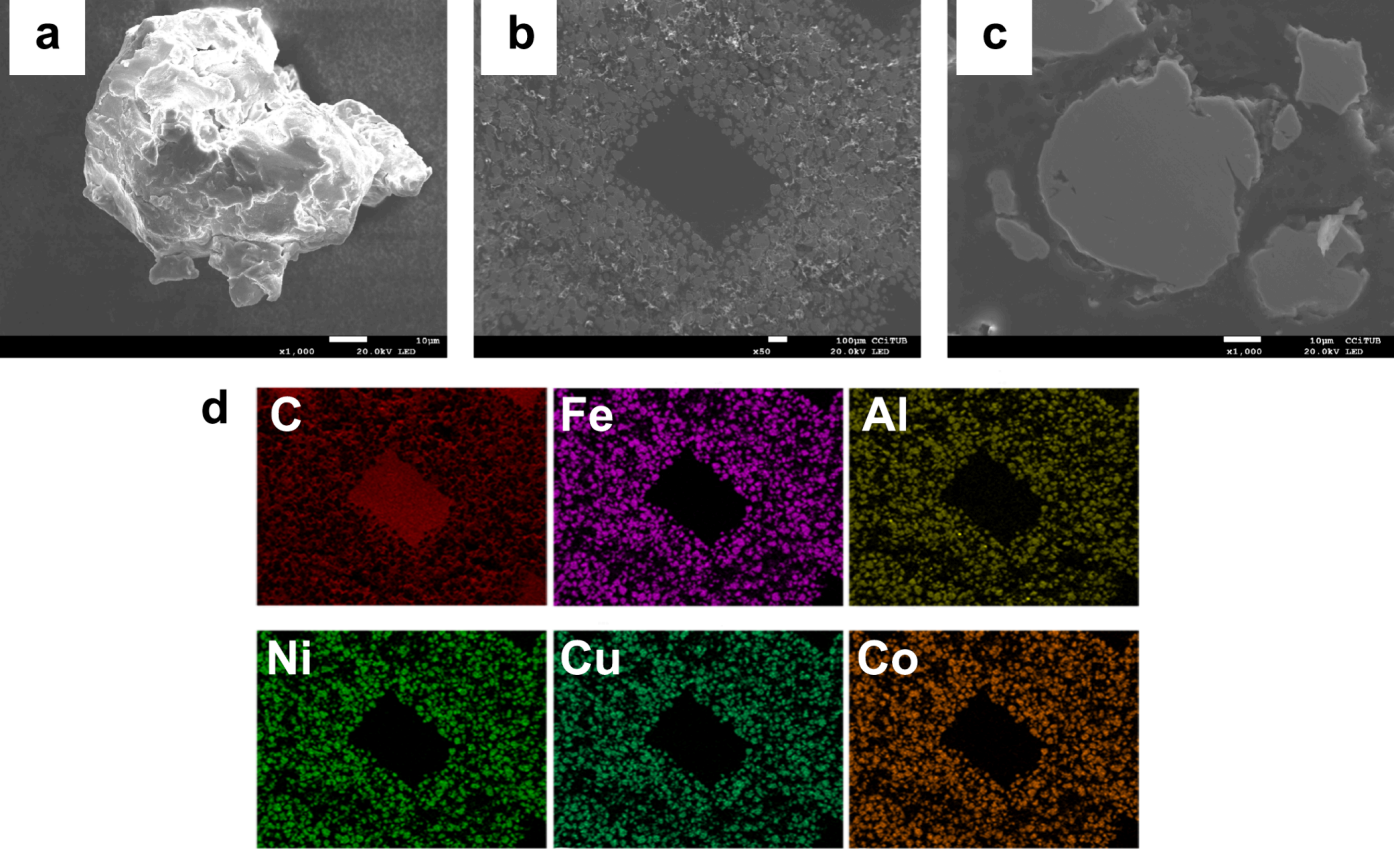
FESEM micrographs EDS compositional map of the achieved HEAs powder after 40 h of HEBM at 300 rpm: (a) single article of the HEA, (b) 3D-printed mesh structure, (c) magnification of the printed structure showing a HEA particle, and (d) EDS compositional map of the particles.

XRD was used to carefully study the evolution of the phases, as well as the achievement of a solid solution during the MA process (Figure 3a). The results reveal that initially, prior to the milling process, the phases corresponding to the metallic powders of iron, aluminum, nickel, copper, and cobalt were present. The first notable change occurs at 1 h of milling, with the disappearance of the aluminum peak, suggesting its incorporation into the crystalline lattice of another element. Additionally, a significant decrease in the intensity of the copper peaks is observed at the same milling time, indicating the beginning of copper’s incorporation into the lattice of another metal. Similarly, there is a drastic reduction in the intensity of the cobalt peak from 0 to 1 h of milling. At 51.8°, the intensity of the nickel peak does not decrease as much as the other elements, suggesting that at short times, all metals in the alloy may enter the nickel crystalline lattice. Between 4 and 20 h of milling, a significant change is observed: at 4 h, two distinct peaks are still visible at 43.2 and 44.6°, whereas at 20 h, only a single peak is present. At 4 h, the nickel peak remains, but shifts toward lower angles, indicating the incorporation of larger atoms into its lattice. Concurrently, the intensity of the copper peak increases compared to 1 h, and a junction zone forms between the peaks at 43.2 and 44.6°. Subsequently, at 20 h, the main peak shifts toward even lower angles, suggesting further changes in the alloy’s crystalline lattice, with lattice parameters resembling those of copper more closely due to the FCC structure. With an increase in milling time to 20 and 40 h, a peak indicative of solid solution emerges, and the dominance of nickel diminishes. Finally, after 40 h of milling, the diffractogram exhibits five peaks corresponding to an FCC crystalline structure with a lattice parameter of *a*_o_ = 3.638 Å. The *hkl* distances (*d_hkl_*) for each angle can be calculated as follows: at 43.1°, *d_hkl_* = 2.09 Å; at 49.9°, *d_hkl_* = 1.82 Å; at 73.7°, *d_hkl_* = 1.28 Å; at 89.3°, *d_hkl_* = 1.09 Å; and at 94.9°, *d_hkl_* = 1.05 Å. TEM analysis was additionally carried out to achieve a more detailed characterization of the microstructure attained for the HEA following 40 h of MA. The bright-field TEM image illustrates finely grained microstructures of the as-milled HEA, with inclusions measuring between 5 and 10 nm (Figure 3b). Figure 3c shows the SAED pattern, which exhibits a characteristic concentric ring structure composed of discrete spots, indicating the polycrystalline nature of the alloy. The bright spots observed in the SAED correspond to diffraction peaks, signifying the presence of well-defined crystal planes within the material. Measurements of the distances between each spot were conducted to determine the interplanar distance. The results yielded interplanar distances of *d_hkl_* = 2.12 Å for (111), *d_hkl_* = 1.87 Å for (200), and *d_hkl_* = 1.30 Å for (220). These findings are in agreement with the distances determined from the XRD data, further confirming the presence of a FCC crystal structure.

**Figure 3:**
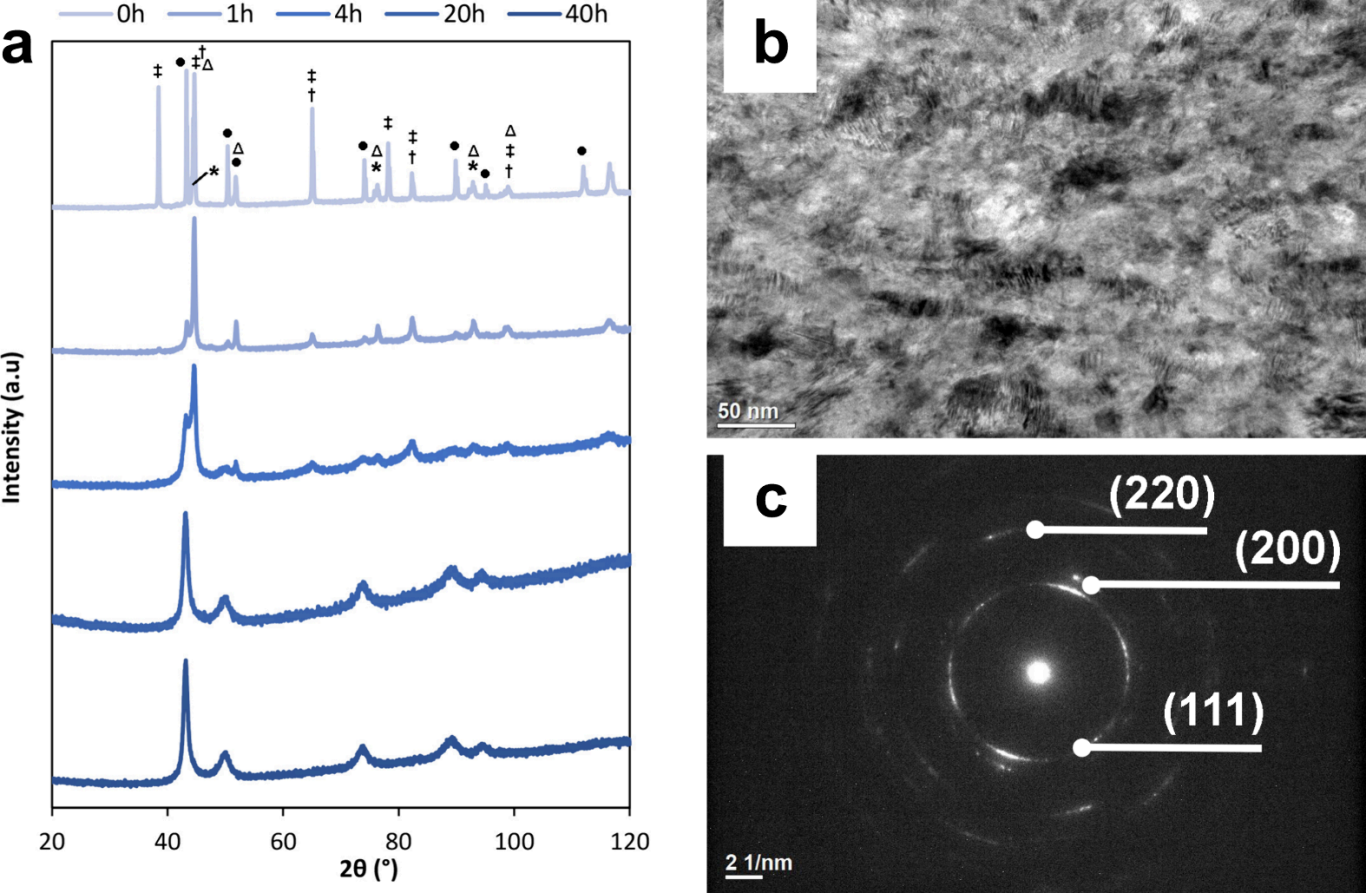
Crystalline structure of the HEA: (a) evolution of the observed phases (Fe (†), Al (‡), Ni (Δ), Cu (•), and Co (*)) with XRD through the MA; (b) bright-field TEM image showing that the HEA consists of nanosized grains; and (c) SAED pattern revealing the polycrystalline nature of the HEA and the corresponding planes to the FCC crystal structure.

ATR-FTIR analysis was used to characterize the chemical composition of the binder used in preparing the composite ink (Figure 4). The ATR-FTIR spectrum revealed the presence of characteristic bands, where δ denotes bending and ν denotes stretching, corresponding to the PLA polymer employed as the binder during the 3D printing process using ME-DIW technology. The presence of PEG was not detected, likely due to its small quantity within the compound. In the region associated with C–H bonds, small bands at approximately 3000 cm^–1^ were observed, assigned to νCH­(sp^3^). In the carboxyl region, a sharp and intense band at approximately 1749 cm^–1^ corresponding to νC=O was identified. In the C–H bending region, two sharp bands of low intensity were noted at approximately 1451 and 1353 cm^–1^, both assigned to δ C–H. Finally, at approximately 1181 and 1080 cm^–1^, sharp bands of medium intensity were observed, assigned to ν C–O.

**Figure 4:**
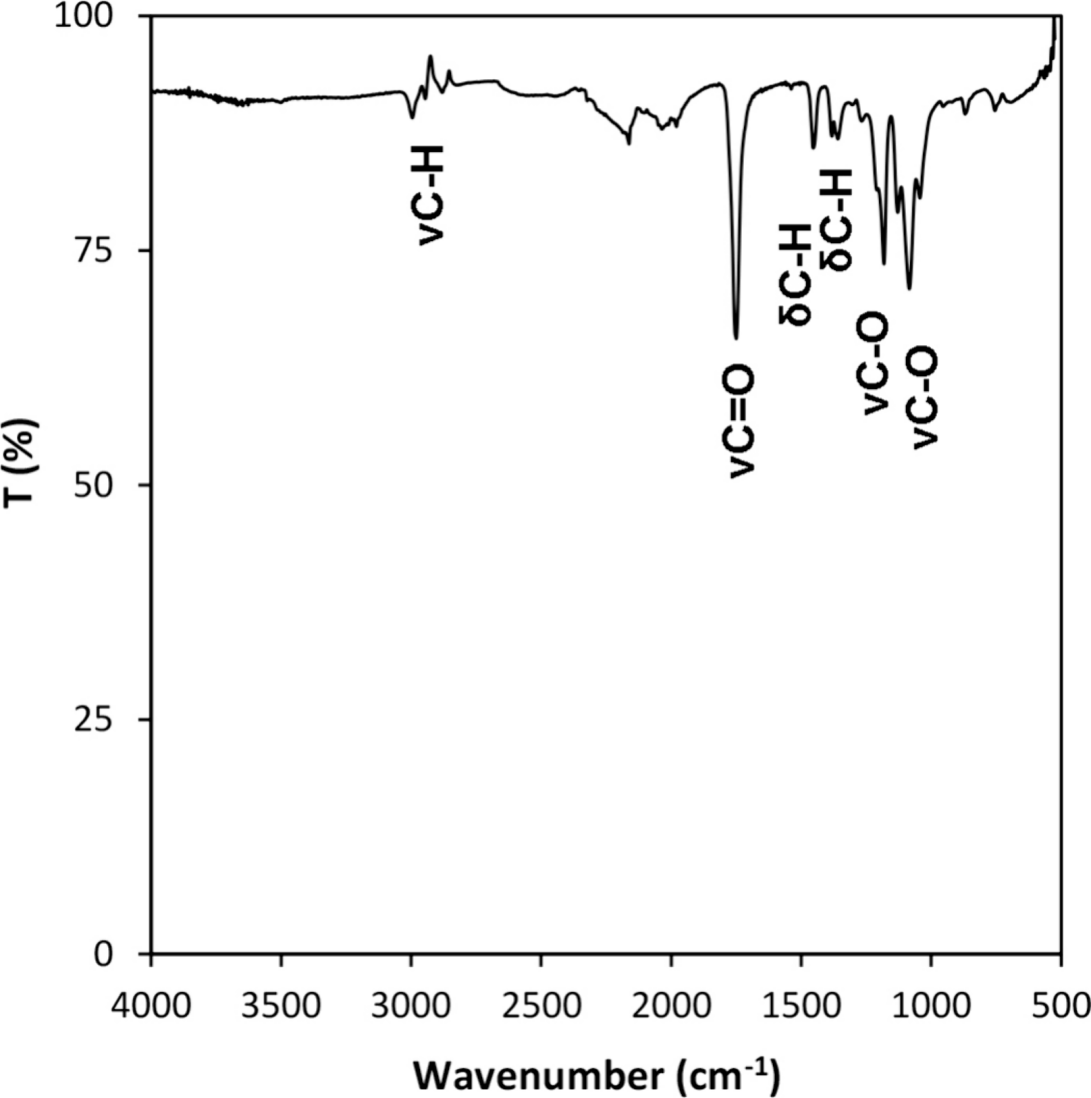
ATR-FTIR spectrum of the PLA–PEG binder used to prepare the metallic-polymeric ink for the 3D printing of the HEA.

The BET analysis enabled measurement of both the specific surface area and pore size of the synthesized HEA (Figure 5). The BET analysis reveals a square root-type function without the presence of a hysteresis loop. The observed isotherm corresponds to Type II, which is associated with the monolayer coverage of the adsorbate due to physisorption.^37,38^ The BET surface area was 0.1741 m^2^/g, while the average pore width for adsorption was 17 Å. This size is typical of micropores (<2 nm) or nonporous materials, where the exposed surface resides almost exclusively within the micropores. Based on the observed results, the prepared material contains micropores or may even be classified as nonporous. Such characteristics suggest that the diffusion of methyl red, considered as a large molecule, into the internal structure is restricted or negligible. As a result, the active sites available for the catalytic process are most likely located on the external surface of the HEA. Consequently, the degradation of the azo dye is expected to occur primarily at the surface level, rather than within internal pores, which may influence both the reaction kinetics and the accessibility of reactive sites.

**Figure 5:**
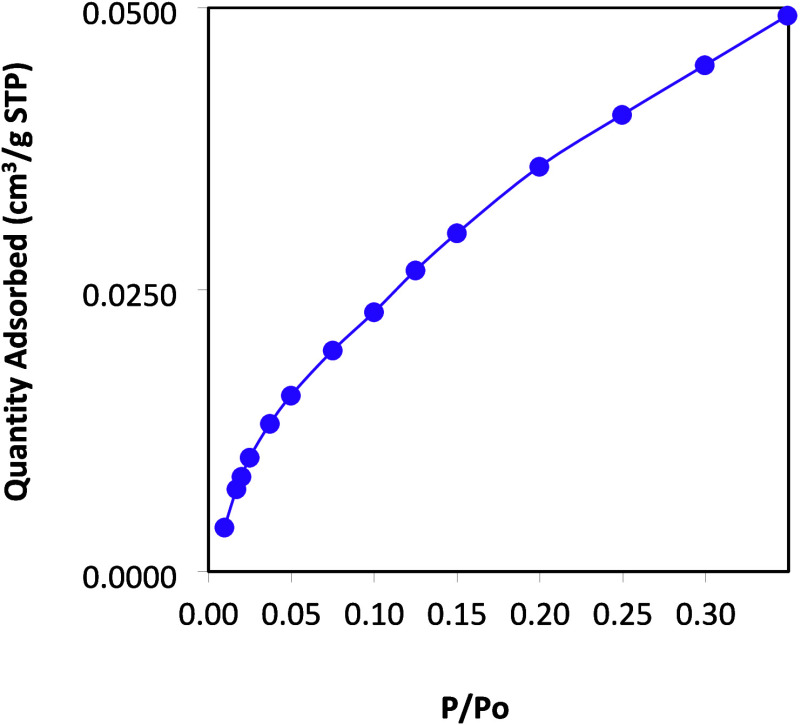
BET analysis for HEAs showing an evolution of the quantity of adsorbed nitrogen characteristic of surface micropores.

### 3.2 Dye Degradation

ME-DIW AM technology was used to 3D print an HEA structure (Figure 6a) for investigating the solid solution ability to degrade methyl red. As depicted in Figure 6b, after 28 min of degradation, the intense peak at 520 nm, corresponding to the azo bond (R–N=N–R’), decreased until no color evidence was detected. In contrast, absorption bands in the 200–300 nm region, associated with phenyl substituents, persisted, indicating that these groups were retained as products of the chemical reaction. This observation indicates that the 3D-printed HEA facilitates degradation by cleaving the azo bond. Furthermore, the degradation process was followed through changes in dye concentration and percentage degradation. On the one hand, the decrease in methyl red concentration was determined according to the Lambert–Beer equation (eq 1):

A=εcl 1

where *A* represents the absorbance at 520 nm, ε is the molar absorption coefficient, *c* denotes the concentration of the dye, and *l* is the length of the cell (10 mm). As illustrated in Figure 6c, the concentration of methyl red decreased from 10 mg/L initially until it reached a minimum value after 28 min of HEA utilization for the degradation process. On the other hand, the percentage degradation can be calculated as follows (eq 2):

%Degradation=(Ao−AtAo)×100 2

where *A*_o_ is the initial measurement of the absorbance and *A_t_* is the absorbance at a specific time (*t*). This expression allows us to determine that the degradation of methyl red using the 3D-printed HEA structure was complete after 28 min, achieving 100% degradation (Figure 6d). Additionally, the pH was continuously monitored during the azo dye degradation process to assess its relevance. As shown in Figure 6e, the pH values increased from the initial value (pH = 1.5) and reached values near 5.5 by the end of the process. This increase in pH indicates that protons were consumed during the degradation of methyl red, which played a crucial role in the mechanism. Notably, two changes in the slope of the pH curves were observed around pH 3.70 and 4.23, suggesting that two consecutive chemical reactions occurred during the degradation process. In the first reaction stage, the pH increase indicates that protons reacted in the aqueous medium until reaching a plateau at pH 4.23, where protons were no longer consumed. Following this, the pH continued to rise rapidly until a second plateau was achieved at pH 5.5, after which the pH value remained constant, indicating that protons were consumed during this second stage and were no longer consumed once this pH was reached. These findings are significant for understanding the surface mechanism occurring at the HEA surface, which is further discussed.

**Figure 6:**
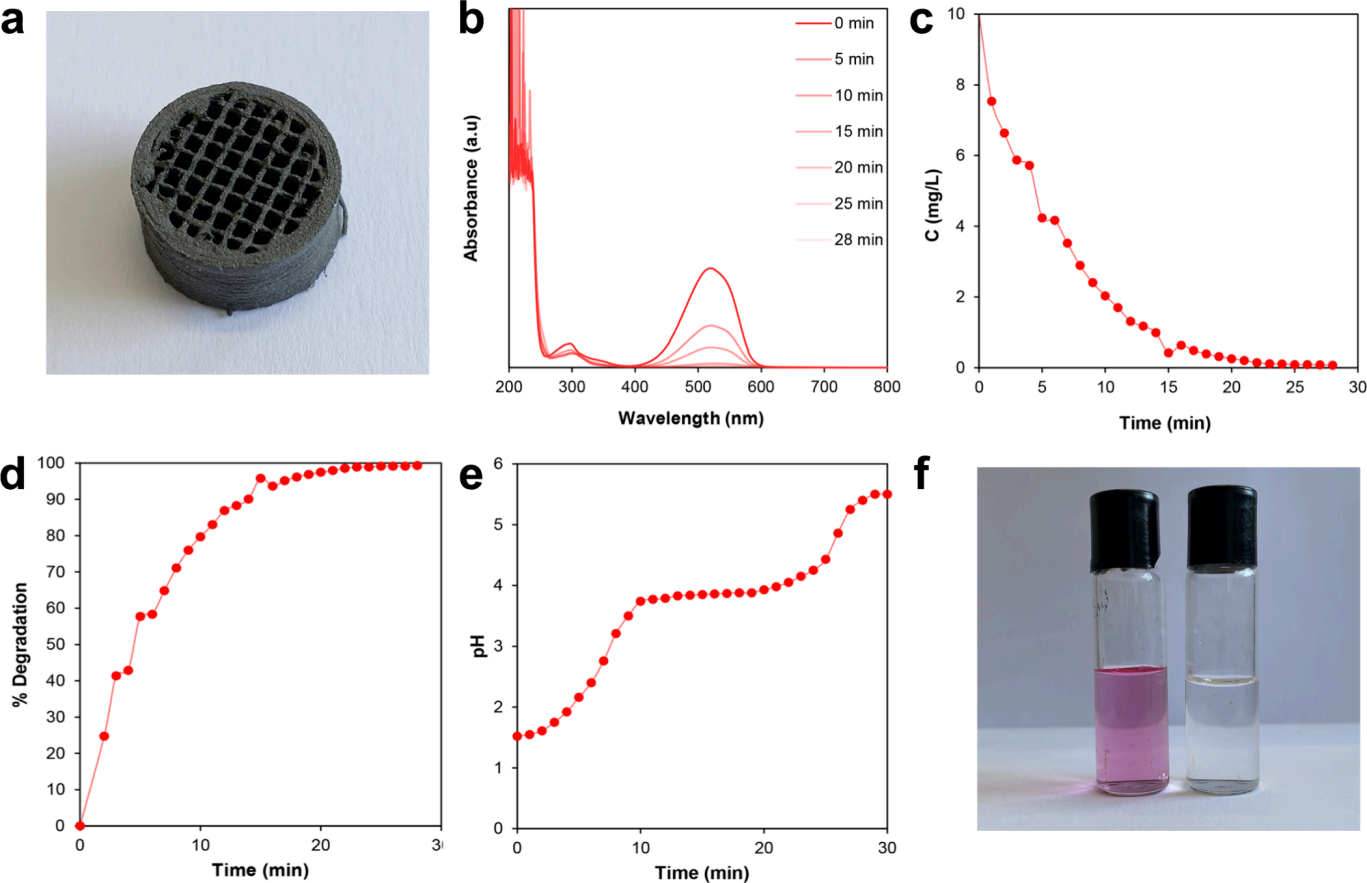
Methyl red degradation process: (a) 3D-printed scaffold, (b) UV–vis spectrophotometry showing that after 28 min, the aqueous solution did not show color, (c) decrease of dye concentration, (d) increase of the degradation of methyl red, (e) evolution of pH, indicating that protons were consumed during degradation, and (f) image showing the degradation of the dye before (left) and after (right) the process.

Additionally, it is essential to determine the key parameters for studying the degradation of methyl red using HEA, including kinetic and thermodynamic parameters such as the reaction constant, half-life time, and activation energy (Table 3). To thoroughly examine the reaction kinetics, the concentration values were linearized (1/*C^n^*) to determine the reaction order (*n*), the kinetic constant (*k*), and the half-life of the reaction (*t*_1/2_). From the general expression of an unknown reaction order (eq 3), the values of *k* and *t*_1/2_ can be determined:

1[A]n−1−1[A]on−1=(n−1)kt 3

where [*A*] is the reactant concentration, [*A*]_o_ is the reactant concentration at the beginning of the reaction (10 mg/L), *k* is the reaction constant rate, and *t* is time. The results indicate that the degradation reaction followed first-order kinetics (*n* = 1), with a reaction rate constant of 0.19 L/mol s and a half-life of 4.11 min, which implies that the reaction rate depends linearly on the concentration of a single reactant, and it is the methyl red. Additionally, the activation energy can be calculated according to the Arrhenius expression (eq 4):

$$k=Ae^{-E_{a}/RT}$$ 4

where *k* is the reaction constant rate, *A* is the pre-exponential factor, *E*_a_ is the activation energy, *R* is the universal gas constant (8.31 J/K mol), and *T* is the absolute temperature in Kelvin. Then, the activation energy for the degradation reaction can be calculated using the linearized form of the Arrhenius equation, as the activation energy is defined to be (−*R*) times the slope of a plot of ln *k* against (1/*T*). Additional experiments were conducted at 20, 40, and 60 °C to determine the reaction rate constant for each temperature. Finally, the activation energy was determined as 25 kJ/mol, revealing that the reaction was endothermic (*E*_a_ > 0). Therefore, it was necessary to heat the system to 60 °C in order to promote dye degradation in the presence of the HEA surface.

**Table 3:** Kinetic and Thermodynamic Parameters for Methyl Red Degradation

*n*	*k* (L/mol s)	*t*_1/2_ (min)	*E*_a_ (kJ/mol)
1	0.19	4.11	25

### 3.3 Identification of the Mechanism

Once the kinetics and thermodynamic characteristics of dye degradation were analyzed, it was also important to identify the products of the chemical reaction in order to propose the chemical reaction mechanism that occurs at the surface of the alloy. For this purpose, HR-HPLC-MS was used to elucidate the reaction mechanism. In the chromatogram shown in Figure 7a, a significant molecule signal can be identified, appearing at 3.89 s. This signal was analyzed using mass spectrometry in positive polarity, enabling the detection of the cations generated as products of methyl red degradation. In the MS spectrum (Figure 7b), the most intense signal corresponding to the base peak was observed at 138 *m*/*z*, while another peak appeared at 120 *m*/*z*, corresponding to a different molecule with a lower mass. The base peak was assigned to the cation of anthranilic acid, one of the substituents of methyl red. Additionally, the peak at 120 *m*/*z*, showing a difference of 18 *m*/*z* from the base peak, corresponds to a molecule fragment caused by the loss of a hydroxyl functional group (−OH) from the carboxylic acid, with a water molecule being detected. Based on the results, anthranilic acid was identified as one of the products of dye degradation. However, since the reaction with methyl red generates a cation, the other molecule is likely the anion from 4-(N,N-dimethylamino)­aniline, which cannot be detected in the positive polarity of mass spectrometry.

**Figure 7:**
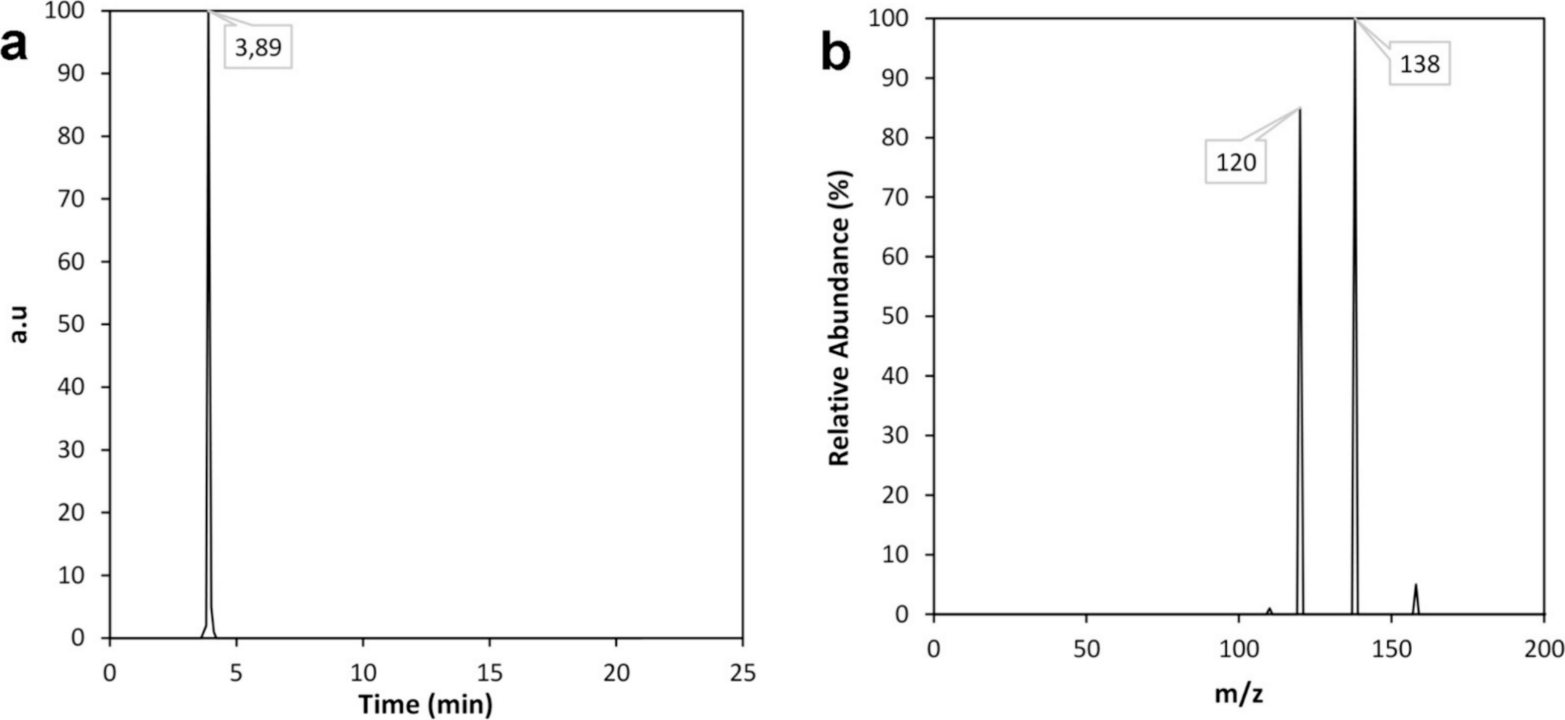
HR-HPLC-MS allows the identification of the reaction product: (a) chromatogram shows a peak at 3.89 s, and (b) MS spectrum displays the cation formed after dye degradation, which is assigned to anthranilic acid.

Based on the results obtained, it can be shown that degradation reactions occurred at the surface of the 3D-printed HEA (Figure 8). Notably, methyl red is a molecule with conjugated double bonds. This structure features alternating double (π) and single bonds, which create a continuous sequence that delocalizes the π electrons through the whole molecule. This delocalization imparts stability to the molecule and gives it its color in aqueous solution, specifically a red color in an acidic medium. During the degradation process, as observed through UV–vis spectrophotometry, the absorbance corresponding to the azo bond at 520 nm decreased from the start, until it was no longer detectable. However, the absorbance bands related to the two phenyl groups in the methyl red molecule remained visible between 200 and 400 nm. Concurrently, the increase in pH during the degradation experiment indicates that protons from nitric acid were consumed in two distinct steps throughout the process. The proposed surface mechanism is described as follows: first, protons and methyl red were adsorbed on the HEA surface and reacted according to the following equation (eq 5):

$$R-N=N-R^{\prime }+2H^{+}\to R-\mathrm{NH}-\mathrm{NH}-R^{\prime }$$ 5

**Figure 8:**
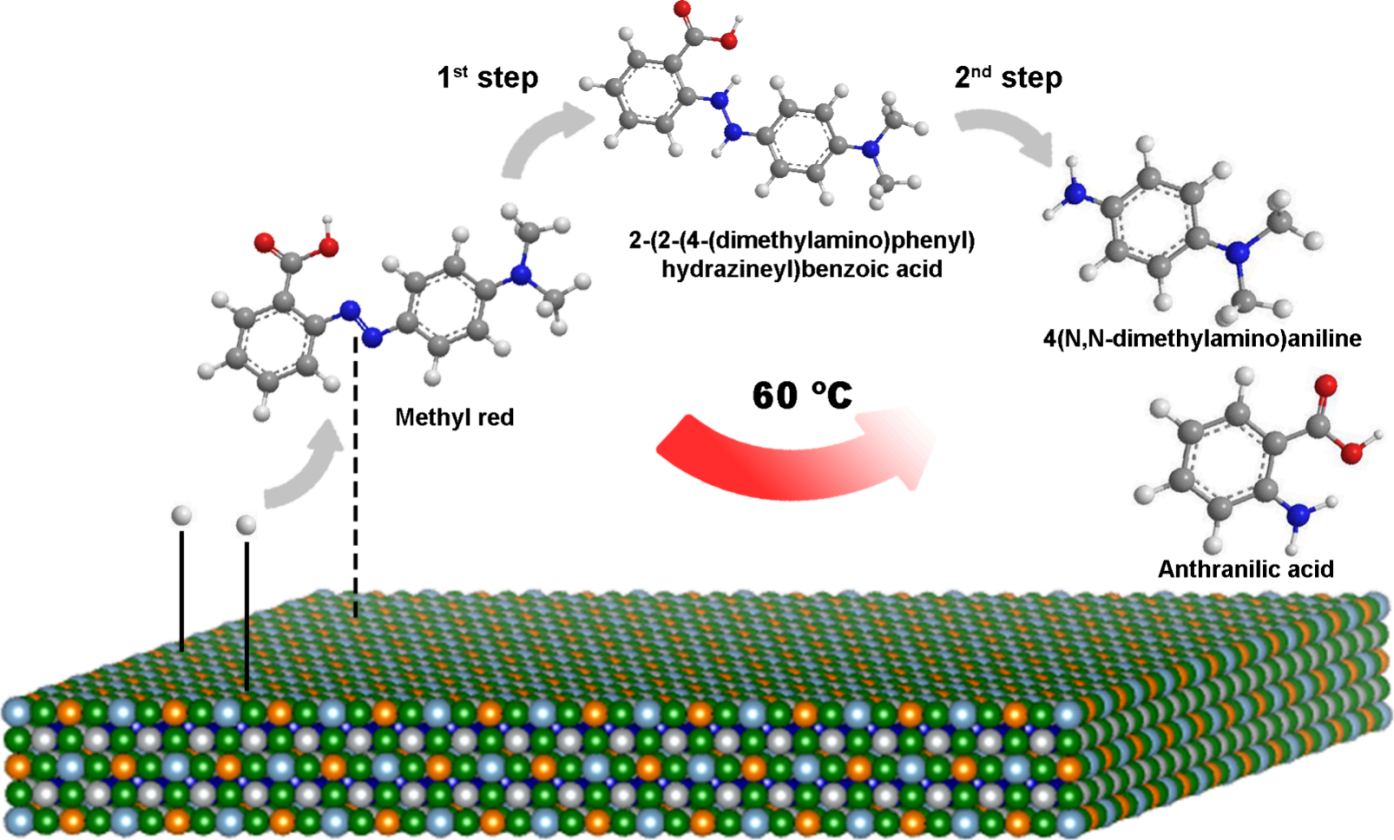
Schematic representation of azo dye degradation with the Fe_27_Al_24_Ni_22_Cu_18_Co_9_ HEA.

Then, as there are still protons in the aqueous solution, they remain adsorbed to the HEA surface, and the new intermediate compound (R–NH–NH–R’) that is 2-(2-(4-(dimethylamino)­phenyl)­hydrazineyl)­benzoic acid reacts with the remaining protons. Once the R–NH–NH–R’ bond is protonated, it breaks, generating two products: anthranilic acid and 4­(N,N-dimethylamino)­aniline (eq 6):

$$R-\mathrm{NH}-\mathrm{NH}-R^{\prime }+2H^{+}\to R-{\mathrm{NH}}_{2}+R^{\prime }-{\mathrm{NH}}_{2}$$ 6

When the azo bond in methyl red becomes protonated and subsequently breaks, the molecular structure of the compound changes. This alteration destabilizes the molecule, disrupting the continuous sequence of double and single bonds and interfering with the delocalization of π electrons. As a result, the aqueous solution loses its characteristic red color, which depends on the specific arrangement of π electrons in the molecule and their interaction with light. In fact, these results are in agreement with the reduction of the azo bond in different kinds of azo dyes.^8,39–41^

## 4 Conclusions

Herein, we show that MA was used to synthesize a Fe_27_Al_24_Ni_22_Cu_18_Co_9_ HEA, achieved after 40 h of milling. Due to its metastable characteristics, the resulting solid solution was utilized in 3D printing with direct ink writing technology, using an ink formulated with metallic particles and PLA as a binder. FESEM analysis demonstrated the homogeneous size and morphology of the prepared metallic powder, while XRD and TEM confirmed the presence of an FCC crystal structure in the HEA.

Subsequently, the environmental application of the solid solution was investigated for the degradation of methyl red, a persistent azo dye. The degradation process proved to be efficient at 60 °C in the presence of nitric acid (pH = 1.50), exhibiting a degradation time of 28 min, a reaction rate constant of 0.19 L/mol s, and an activation energy of 25 kJ/mol. The degradation of the azo dye took place at the surface of the 3D-printed HEA that allowed production, according to the HR-HPLC-MS results, of two different compounds. In overall terms, the achieved Fe_27_Al_24_Ni_22_Cu_18_Co_9_ HEA demonstrates as a promising material for the degradation of azo dyes.

## Supplementary Material



## Notes

Preliminary test of 3D printing showing initial print parameters and specimen morphology (PDF)
