# Supplementary material for: 3D Printing of Fe27Al24Ni22Cu18Co9 High-Entropy Alloy Scaffold with Direct Ink Writing for the Degradation of Methyl Red Azo Dye
Source: ACS Omega. 2025 Oct 30;10(44):52612–20. doi: 10.1021/acsomega.5c06103 (PMC12612945; doi:10.1021/acsomega.5c06103)
Supplement: Supplementary file 1 [file ao5c06103_si_001.pdf]

Supporting Information

3D printing of $Fe_{27}Al_{24}Ni_{22}Cu_{18}Co_9$ high-entropy alloy scaffold with Direct Ink Writing for the degradation of methyl red azo dye

Oriol Rius-Ayra*, Alisiya Biserova-Tahchieva, Marina Carmona-Ruiz and Núria Llorca-Isern

CMQF Departament de Ciència dels Materials i Química Física, Universitat de Barcelona, Martí i Franquès 1-10, 08028, Barcelona, Spain.

*Corresponding author: oriolriusayra@ub.edu

Preliminary test of 3D printing

**a**	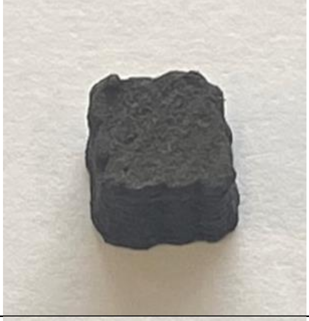	Size (h x w x l): 5 mm x 5 mm x 5mmm<br>Solid structure
**b**	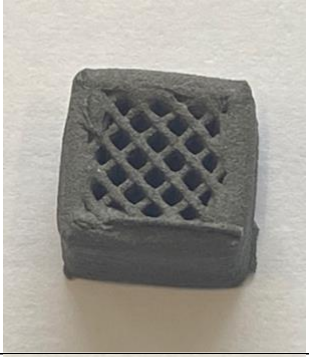	Size (h x w x l): 7 mm x 10 mm x 10 mm<br>Wall = 3 mm<br>Infill 20 %
**c**	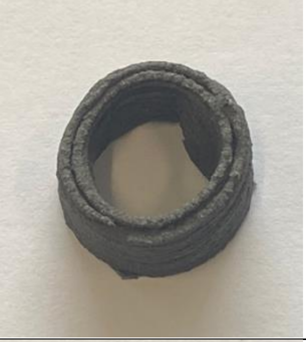	Size: heigh = 10 mm; ∅ = 16 mm<br>Wall = 3 mm<br>No infill
**d**	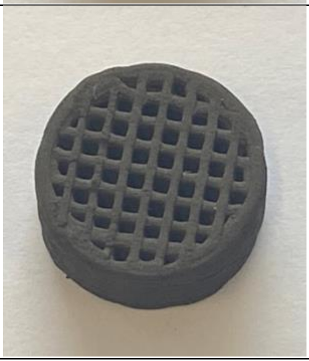	Size: heigh = 10 mm, ∅ = 16 mm<br>Wall = 3 mm<br>Infill = 20 %

Figure SI 1. Different structures and their parameters tested in previous 3D printing experiments.

Before printing the final scaffold structure shown in the manuscript (Figure 1c), a preliminary study was carried out to determine the optimal printing parameters and to avoid printing errors or structural breakage. The parameters considered were: (i) the amount of metallic particles, (ii) the infill, and (iii) the geometry of the structure.

Firstly, regarding the amount of HEA particles, four different compositions of HEA particles with PLA as binder were tested. Structures containing 75 %, 80 %, and 85 % of metallic particles were robust and showed neither breakage nor printing defects. However, when the amount was increased to 90 %, the solid structure broke and could not be printed successfully. Therefore, 85 % of metallic particles was selected as it provided the highest particle content while maintaining robustness.

Secondly, a simple solid cubic geometry was printed successfully, which allowed for an increase in structural complexity (Figure SI 1a). Three different infill values were tested: 10 %, 20 %, and 30 %. It was observed that the 30 % infill could not be printed due to defects, leading to the conclusion that 20 % was the most suitable parameter.

Finally, once the particle content and infill parameters were determined, the structure was further modified to achieve higher complexity. After the initial solid cubic structure, a cube with 20 % infill was successfully printed (Figure SI 1b). The geometry was then modified to a hollow cylinder with a wall thickness of 1.5 mm (Figure SI 1c). Since these previous attempts were successful, the final scaffold structure was ultimately printed (Figure SI 1d).
